# A Lectin-Based Glycomic Approach to Identify Characteristic Features of *Xenopus* Embryogenesis

**DOI:** 10.1371/journal.pone.0056581

**Published:** 2013-02-14

**Authors:** Yasuko Onuma, Hiroaki Tateno, Shingo Tsuji, Jun Hirabayashi, Yuzuru Ito, Makoto Asashima

**Affiliations:** 1 Research Center for Stem Cell Engineering, National Institute of Advanced Industrial Science and Technology (AIST), Tsukuba Central 4, Tsukuba, Japan; 2 Research Center for Stem Cell Engineering, National Institute of Advanced Industrial Science and Technology (AIST), Tsukuba Central 2, Tsukuba, Japan; 3 Genome Science Division, Research Center for Advanced Science & Technology, The University of Tokyo, Tokyo, Japan; University of Colorado, Boulder, United States of America

## Abstract

Cell surface glycans show dynamic changes during cell differentiation. Several glycans are useful biomarkers of tumors, stem cells, and embryogenesis. Glycomic studies have been performed using liquid chromatography and mass spectrometry, which are powerful tools for glycan structural analysis but are difficult to use for small sample sizes. Recently, a lectin microarray system was developed for profiling cell surface glycome changes to terminal carbohydrate chains and branch types, using sample sizes of a few micrograms. In this study, we used the lectin microarray system for the first time to investigate stage-specific glycomes in *Xenopus laevis* embryos. Unsupervised cluster analysis of lectin microarray data indicated that glycan profiles changed sequentially during development. Nine lectin probes showed significantly different signals between early and the late-stage embryos: 4 showed higher signals in the early stages, and 5 exhibited higher signals in the late stages. The gene expression profiles of relevant glycosyltransferase genes support the lectin microarray data. Therefore, we have shown that lectin microarray is an effective tool for high-throughput glycan analysis in *Xenopus* embryogenesis, allowing glycan profiling of early embryos and small biopsy specimens.

## Introduction

Changes in glycan expression are thought to be important in a wide range of bioprocesses, although little is known about the nature and magnitude of glycome changes that occur during embryogenesis.

Here, we examined *Xenopus laevis* glycome profiles during developmental progression using a lectin microarray system, and in parallel, we analyzed the expression profiles of genes related to glycan synthesis using DNA microarray. Lectin microarray is a high-throughput analytical method based on glycan binding to lectins arrayed on a glass slide [1], and this method has been used to search for diagnostic biomarkers [2]. The lectin microarray system revealed stage-specific glycan profiles in *Xenopus* embryos. Significant changes were detected in lectin probe signals, indicating the presence of specific glycans during embryogenesis. The lectin microarray data revealed that the expression of ABO blood-group glycans, ßGalNAc, and αGalNAc (Tn-antigen) decreased during embryogenesis, while the expression of high-mannose–type N-glycans increased. Consistent with this data, the expression of *α1,2 fucosyltransferase* (*fut1*) and *α-N-acetylgalactosaminyltransferase/α1,3 galactosyltransferase* (*abo*) genes, involved in the synthesis of ABO blood-group glycans, was higher in early-stage embryos than at later stages. The UDP-GalNAc:polypeptide *N*-acetylgalactosaminyltransferase genes *galnt3*, *galnt7*, and *galnt11* were highly expressed at early embryonic stages, and their expression gradually decreased at later stages. The reduced expression of *mannoside acetylglucosaminyltransferase 1* (*mgat1*) was consistent with increased high-mannose–type N-glycans during later developmental stages. This study is the first to demonstrate the application of a lectin microarray system in *Xenopus* embryos.

## Materials and Methods

### Ethics Statement

This study was carried out in strict accordance with the National Institute of Advanced Industrial Science and Technology (AIST) guidelines for life science experiments, and all animal experiments were approved by the AIST (accreditation number 2011–109G).

### Animals

Standard *Xenopus* techniques were followed to induce ovulation, perform in vitro fertilization, and dejelly eggs [3]. Embryos were staged according to Nieuwkoop and Faber [4], and samples from 10 unfertilized eggs and embryos at each of the 12 developmental stages from 2–40 were collected for DNA microarray and lectin microarray analysis.

### Lectin microarray and analysis

The design and use of the lectin microarray was described previously (Table S1) [5], [6]. Briefly, triplicate probes of 96 lectins at 0.5 mg/mL were spotted onto glass slides. Embryo samples were homogenized in modified RIPA buffer [7], and clarified supernatants were obtained after centrifugation using a conventional protein extraction procedure. The extracts were labeled with Cy3-*N*-hydroxysuccinimide (NHS) ester (GE Healthcare), diluted to 0.5 µg/ml, and incubated with the lectin microarray at 20°C overnight. Fluorescence images were acquired using an evanescent-field activated fluorescence scanner (GlycoStation Reader 1200; GP Biosciences). The fluorescence signal of each spot was quantified using an Array-Pro Analyzer ver.4.5 (Media Cybernetics), and a background value obtained from an area without lectin immobilization was subtracted from each spot. Lectin signals from triplicate spots were averaged and normalized to the mean value of the 96 lectins in the array.

Unsupervised clustering was performed by the average linkage method using Cluster 3.0 software. The heat map with clustering was obtained using Java TreeView. Differences between 2 arbitrary data sets were evaluated by applying Student's *t*-test to each lectin signal using SPSS statistics 19 (SPSS).

### DNA microarray and analysis

Total RNA was extracted from frozen samples of each embryonic stage using ISOGEN (NIPPON GENE) according to the manufacturer's instructions. Samples were analyzed by Agilent Frog Microarray 4×44 k (G2519F, Agilent Microarray Design ID 015066) using the Quick-Amp Labeling Kit, one color (Agilent). Arrays were scanned using a G2505C Microarray Scanner System (Agilent). Raw microarray data were submitted to the Gene Expression Omnibus (GEO) microarray data archive (http://www.ncbi.nlm.nih.gov/geo/) at the NCBI (accession numbers GSE40620).

Data were analyzed using Gene-Spring GX12.0 software (Agilent) after applying 2 normalization procedures: (1) signal intensities of <1 were set to 1, and (2) each chip was normalized to the 75th percentile of all measurements from that chip. Baseline transformation of those data was not performed. Genes with a flag value of “detected” or “not detected” in at least 1 sample were analyzed. “*Xenopus* glycogenes” gene sets are listed in Table S2 (see text). A heat map in which each square element indicates the correlation coefficient value between 2 developmental stages was acquired using matplotlib (http://matplotlib.sourceforge.net/).

## Results and Discussion

### Comparative glycomics during *Xenopus laevis* development

**Figure 1:**
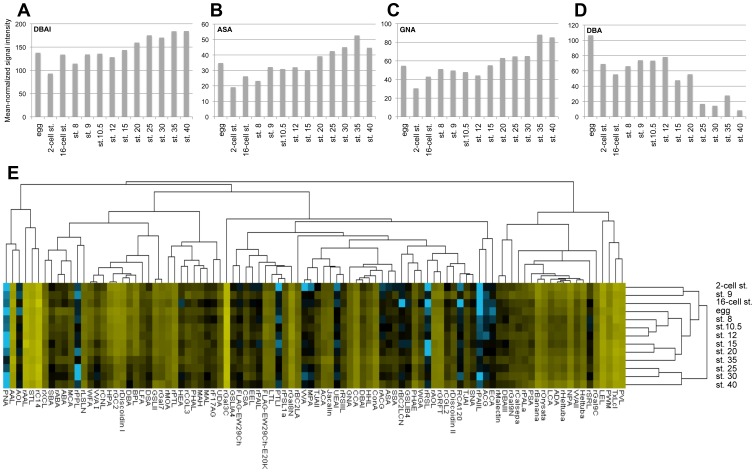
Lectin microarray data of *Xenopus* embryogenesis. (A–D) Lectin microarray data for the 4 indicated lectins, DBAI (A), ASA (B), GNA (C), and DBA (D). Mean-normalized signal intensities of lectins are shown. All lectin data are shown in Table S3. (E) A summary of 96 lectin signals. Unsupervised hierarchical cluster was generated using mean-normalized and log-transformed signal intensities. Levels of lectin signaling are indicated by a color change from blue (low) to yellow (high). Egg, unfertilized egg; st., stage.

To compare developmental stage profiles of protein glycomics during *Xenopus* embryogenesis, we analyzed unfertilized eggs and 12 embryonic development stages: blastomere (2-cell and 16-cell stages), blastula (stages 8 and 9), gastrula (stages 10.5 and 12), neurula (stages 15 and 20), tailbud (stages 25, 30, and 35), and tadpole (stage 40; Table S1). For this, crude protein extracts were labeled with the Cy3-*N*HS ester and analyzed by lectin microarray (Figure 1; Table S3). Mean-normalized signal intensity data were first analyzed by unsupervised hierarchical clustering (Figure 1E). The heat map comprises a matrix showing distances between each developmental stage (Figure 1E). We found that contiguous developmental stage embryos have similar glycan profiles, indicating that glycan profiles change gradually throughout development. The representative examples of 4 lectins are consistent with the gradually increasing or decreasing glycan profiles during embryogenesis (Figure 1A–D).

### Characterization of glycome changes during embryogenesis

To examine developmental transcriptome features related to the glycan profiles, we used DNA microarray to obtain gene expression profiles of sibling embryos used for lectin microarray. We listed as “*Xenopus* glycogenes” those genes related to glycan synthesis, including glycotransferases, sulfotransferases that add sulfate to carbohydrates, sugar-nucleotide transporters, and putative glycotransferase genes identified by a homology search of databases including a human glycogene library (http://riodb.ibase.aist.go.jp/rcmg/ggdb/) [8], Xenbase (http://www.xenbase.org/) [9], NCBI (http://www.ncbi.nlm.nih.gov/), and other published information (Table S2).

**Figure 2:**
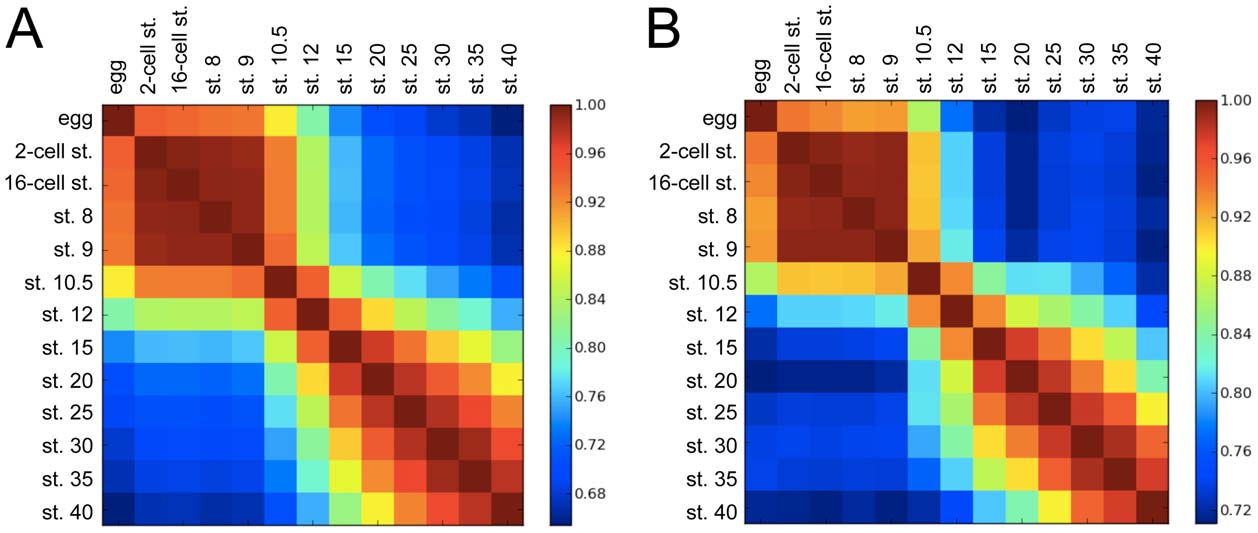
Global comparisons of total and glycogene transcriptomes during *Xenopus* embryogenesis. Heat map of the correlation coefficient matrix. The color of each square indicates the difference at a specified developmental stage. By definition, diagonal squares have zero divergence. Color scales indicate the correlation coefficient. (A) Global genes; (B) “*Xenopus* glycogenes.” Egg, unfertilized egg; st., stage.

The correlation coefficient matrix for gene expression at different developmental stages shows that changes in global gene expression do not follow a smooth continuum (Figure 2A). Three gene clusters appear to define distinct transcriptional states: early stage (2-cell stage, 16-cell stage, stage 8, and stage 9), gastrula (stages 10.5 and 12), and late stage (stages 15–40). The early-stage gene cluster shares some maternal gene expression with unfertilized eggs, and the late-stage gene cluster shows gradual changes in the transcriptome during developmental progression (Figure 2A). In addition, the gastrula stage (stages 10.5 and 12) is distinguishable from these 2 groups. Changes in global gene profiles may reflect multiple differentiation-related changes that occur during gastrulation. Interestingly, the same clusters were observed in the correlation coefficient matrix for “*Xenopus* glycogenes” (Figure 2B). These significant changes in the glycogene transcriptome suggest that dynamic changes in glycan expression occur during gastrulation.

**Figure 3:**
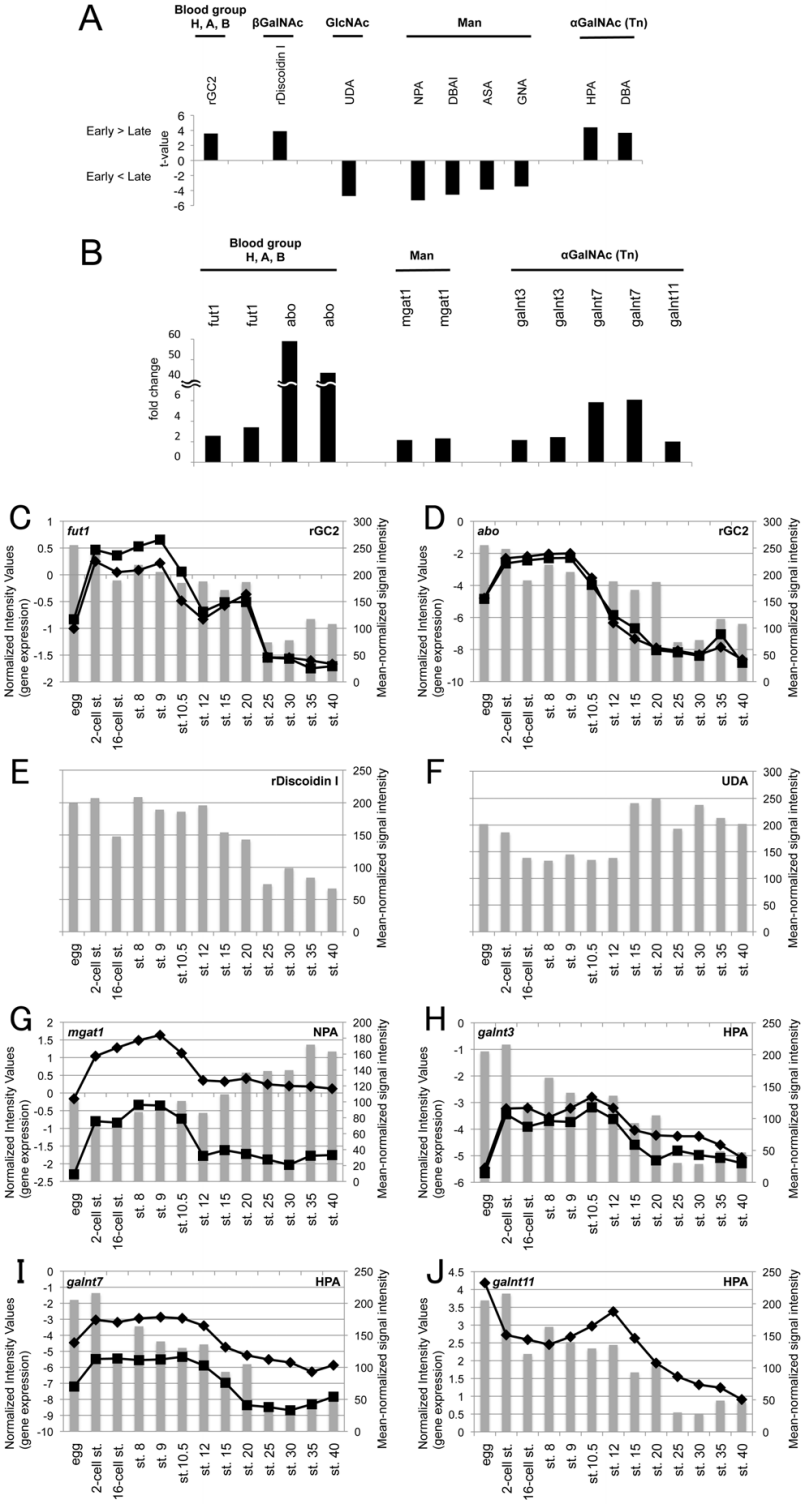
Alterations to glycan and glycogene expression during embryogenesis. (A) Lectin microarray data were mean-normalized and analyzed by Student's *t*-test. Nine lectins with significantly different signals (*p*<0.01) between the early-stage group (2-cell stage, 16-cell stage, stage 8, and stage 9) and the late-stage group (stages 15–40) were categorized into 5 groups based on the glycan-binding specificities of lectins (indicated above). *t*-Values of lectins with higher signals in the early-stage group than in the late-stage group (indicated as Early>Late) are displayed as positive values, and *t*-values of lectins with lower signals in the early-stage group (indicated in Early<Late) are displayed as negative values. All lectin data are shown in Table S1. (B) Changes in the expression of glycogenes related to lectins with significantly different signals in early- and late-stage groups. Fold changes in 11 DNA microarray probes for glycogenes and glycans that may be regulated by these glycogenes are shown. All gene data are deposited in the GEO database as GSE40620. (C–J) Temporal changes in lectin signals and glycogene expression during embryogenesis. Normalized intensity values of glycogene probes are represented as lines in the primary Y-axis (left side). Mean-normalized signal intensities of lectins are represented as bars in the secondary Y-axis (right side). All lectin data are shown in Table S3. (C) *fut1* and rGC2, (D) *abo* and rGC2, (E) rDiscoidin I, (F) UDA, (G) *mgat1* and NPA, (H) *galnt3* and HPA, (I) *galnt7* and HPA, (J) *galnt11* and HPA.

**Figure 4:**
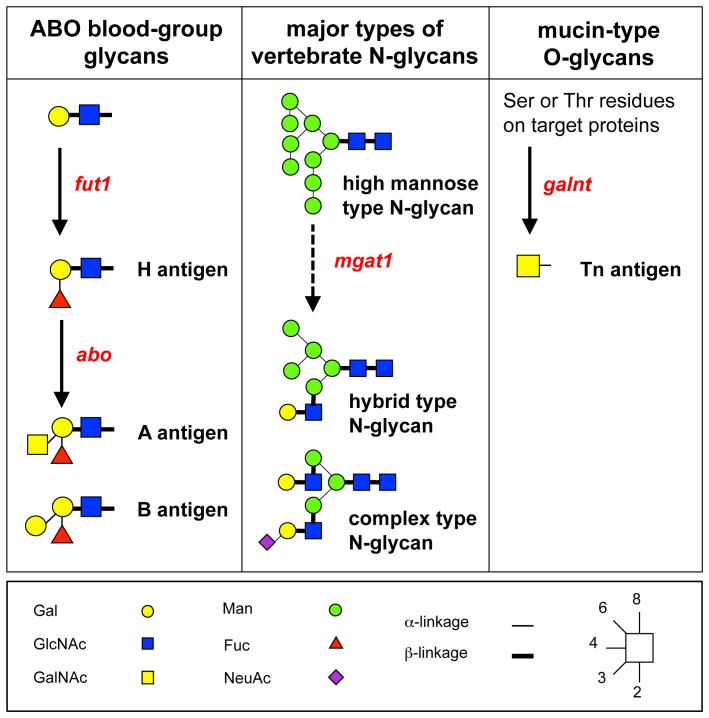
Schematic relationships between the glycans and transferase genes, whose expressions differed significantly between early- and late-stage embryos in this study. Glycosyltransferase genes are indicated in red. The *mgat1* gene does not directly produce hybrid type or complex type of N-glycans, but a high expression level of *mgat1* reduces levels of high-mannose-type N-glycans and increases the production of hybrid and complex-type N-glycans.

We next identified glycome alterations occurring during embryogenesis. Mean-normalized data were analyzed using Student's *t*-test to select probe lectins that differed significantly between early-stage (2-cell stage, 16-cell stage, stage 8, and stage 9) and late-stage (stages 15–40) embryos (Table S4). As a result, 9 lectins were selected for which *p*<0.01; 4 showed higher signals in the early-stage group than the late-stage group, and 5 exhibited lower signals (Figure 3A; Table S4). The 9 lectins were classified into 5 groups based on their glycan-binding specificities (Figure 3A). The sequential changes in lectin signals (Figure 1A–D; Figure 3A–H) provided characteristic features of glycan structures during embryogenesis: (1) rGC2 signals specific to blood groups A and B were higher in early-stage embryos than in late-stage embryos (Figure 3C,D); (2) signals of rDiscoidin I, which binds to βGalNAc, were higher in early stages than in late stages (Figure 3E); (3) signals of the GlcNAc-binding lectin UDA were increased in later-stage embryos (Figure 3F); (4) signals of a series of mannose-binding lectins (NPA, DBAI, ASA, and GNA) were increased in later-stage embryos (Figure 1A–C; Figure 3G); and (5) signals of the αGalNAc (Tn-antigen)-binding lectins, HPA and DBA, were higher in early-stage embryos (Figure 1D; Figure 3I, J).

### Glycosyltransferase expression profiles alter during embryogenesis

We next investigated the expression of the glycosyltransferase genes associated with lectins that showed characteristic changes during developmental progression (Figure 3B). *fut1* and *abo* expressions were higher in early-stage embryos than in late-stage embryos, thus corresponding to the rGC2 expression pattern (Figure 3C,D). The *fut1* gene regulates expression of H antigen, and *abo* glycosyltransferases attach α1,3GalNAc or α1,3Gal onto the H antigen in the human erythrocytes, endothelia, and epithelial cell membranes (Figure 4). Previous reports indicate that blood group B active membrane glycoproteins participate in Ca^2+^-dependent cell–cell adhesion in *Xenopus* blastula cells [10], [11]. Our data from both lectin and DNA microarrays indicate that B blood-group glycans are present in the *Xenopus* blastula (stages 8–9).

The expression of *mgat1*, essential for the synthesis of complex- and hybrid-type N-glycans, was high at early embryonic stages, decreased from stage 10.5 onwards, and was low at stage 12 and later (Figure 3G). *mgat1* expression results in the production of complex, hybrid N-glycans and reduces levels of high-mannose–type *N*-glycans (Figure 4). Consistent with this, the signals of high-mannose–type *N*-glycan-binding lectins (NPA, DBAI, ASA, and GNA) increased from stage 15 onwards (Figure 1A–C; Figure 3G).

*UDP-GalNAc:polypeptide N-acetylgalactosaminyltransferase* genes (*galnt3*, *galnt7*, and *galnt11*) were highly expressed at early embryonic stages and gradually reduced from stage 12 to 15 (Figure 3H–J). In addition, αGalNAc (Tn-antigen) expression, as indicated by HPA and DBA signals, was higher in early stages than in later stages (Figure 1D; Figure 3H–J). GALNT transferases synthesize mucin-type *O*-glycans by transferring αGalNAc to Ser or Thr residues on targets proteins (Figure 4) [12]. Structural changes to these *O*-linked glycans can affect normal bioprocesses and diseases, including cancer metastasis [13]. A recent study suggested that *galnt11* is a candidate gene for congenital heart disease caused by heterotaxy [14]. Knockdown of *Xenopus galnt11* causes the disruption of left–right body patterning; however, the timing and mechanism for the requirement of *galnt11* activity and *O*-linked glycans are unclear [14]. Our results that both *galnt11* expression and *O*-linked glycans are highly expressed during early embryonic stages and gradually decrease may provide insight into these processes.

In this study, we examined developmental stage-specific glycan profiles in *Xenopus* embryos. Lectin microarray indicated that changes to glycan profiles were sequential. We observed that blood groups A and B, βGalNAc, and αGalNAc (Tn-antigen) decreased, while high-mannose–type N-glycans increased, during developmental progression. The gene expression profiles of associated glycosyltransferase genes are consistent with the glycan profiles obtained by lectin microarray. As development progresses, the number of distinct embryonic domains increases, so that the overall global glycome patterns in embryogenesis necessarily present an oversimplified description and are likely to obscure regional changes. However, this is the first study to apply the lectin microarray system to analyze *Xenopus* embryo glycome. We have shown that the lectin microarray system has the potential for application in high-throughput glycan analysis in a wide range of biological processes.

## Supporting Information

Table S1 **Identity of lectins used in the lectin microarray.**
(XLS)

Table S2 ***Xenopus*** **glycogenes.**
(XLS)

Table S3 **Lectin microarray signal intensities for each developmental stage.**
(XLS)

Table S4 **Comparison of the lectin signals between early and late developmental stages.**
(XLS)
